# The quasi-parallel lives of satellite cells and atrophying muscle

**DOI:** 10.3389/fnagi.2015.00140

**Published:** 2015-07-22

**Authors:** Stefano Biressi, Suchitra D. Gopinath

**Affiliations:** ^1^Dulbecco Telethon Institute and Centre for Integrative Biology (CIBIO), University of TrentoTrento, Italy; ^2^Translational Health Science and Technology Institute (THSTI)Faridabad, India

**Keywords:** skeletal muscle, stem cells, satellite cells, atrophy, cachexia

## Abstract

Skeletal muscle atrophy or wasting accompanies various chronic illnesses and the aging process, thereby reducing muscle function. One of the most important components contributing to effective muscle repair in postnatal organisms, the satellite cells (SCs), have recently become the focus of several studies examining factors participating in the atrophic process. We critically examine here the experimental evidence linking SC function with muscle loss in connection with various diseases as well as aging, and in the subsequent recovery process. Several recent reports have investigated the changes in SCs in terms of their differentiation and proliferative capacity in response to various atrophic stimuli. In this regard, we review the molecular changes within SCs that contribute to their dysfunctional status in atrophy, with the intention of shedding light on novel potential pharmacological targets to counteract the loss of muscle mass.

## Introduction

Skeletal muscle atrophy is characterized by a loss of muscle mass and force, that occurs in response to a variety of pathological and physiological stimuli such as aging, cancer, chronic kidney disease (CKD), chronic obstructive pulmonary disease (COPD), chronic heart failure (CHF), diabetes, AIDS infection, sepsis, burns, muscle disuse, loss of muscle innervation, malnutrition, steroid-induced catabolic stimulation, and different congenital neuromuscular diseases (Bonaldo and Sandri, 2013; Egerman and Glass, 2014; Cohen et al., 2015). Loss in muscle mass is characterized by a decrease in cross-sectional area of muscle fibers, that is primarily the outcome of a preferential increase in intracellular protein degradation over protein synthesis (Bonaldo and Sandri, 2013; Egerman and Glass, 2014; Cohen et al., 2015). In most muscle wasting conditions, the loss of muscle tissue is not homogeneous, displaying differential effects on specific muscle groups and impacting distinct fiber types (Ciciliot et al., 2013). In specific forms of atrophy, such as age-related atrophy (sarcopenia), the reduction in fiber size is also accompanied by a reduction in the number of fibers, further highlighting the heterogeneity of the atrophying process occurring in different muscle wasting conditions (Lexell et al., 1988).

Muscle fibers are post-mitotic syncytia formed by the fusion of several hundreds of myogenic progenitors during pre- and post-natal development (Biressi et al., 2007). A majority of the myonuclei are added to developing fibers during post-natal growth, when there is a dramatic increase in muscle mass (Zhang et al., 1998). Not all myogenic progenitors terminally differentiate into muscle fibers during development, but a fraction of them remains in the adult muscles as a pool of undifferentiated myogenic stem cells. Different cellular types that possess the ability to differentiate into muscle fibers have been identified (Cossu and Biressi, 2005; Peault et al., 2007). Nevertheless, a population of stem cells, called “satellite cells” (SCs), due to the peculiar anatomical location between the fiber sarcolemma and the basal lamina surrounding the fiber, is believed to contribute in a major way to post-natal growth and muscle repair upon injury (Schultz, 1996; Lepper et al., 2011; Murphy et al., 2011; Sambasivan et al., 2011). In healthy adult muscles, SCs are largely quiescent, but can be activated by appropriate stimuli in the form of injury or exercise, resulting in regeneration and possibly further growth (hypertrophy) of the muscle tissue (Zammit et al., 2006; Schiaffino and Partridge, 2008). Several studies have investigated the role played by SCs in muscle hypertrophy and conclusions drawn from these studies are equivocal (Blaauw and Reggiani, 2014). A key role for SCs in the hypertrophic response is supported by observations that SCs are consistently activated in hypertrophic models and that their elimination by irradiation severely blunts overload-induced muscle hypertrophy (Rosenblatt et al., 1994). On the contrary, various observations using mutant mice to genetically ablate SCs and induce hypertrophy indicate that SC functionality does not constitute an absolute requirement in initiating muscle hypertrophy, suggesting that the prerequisite nature of their involvement might be limited to later stages in stabilizing the full hypertrophic response on a long-term basis (Amthor et al., 2009; Blaauw et al., 2009; McCarthy et al., 2011; Lee et al., 2012; Fry et al., 2014).

Whereas our knowledge of SC behavior in muscle hypertrophy is rapidly increasing, the role played by SCs during muscle atrophy remains largely enigmatic. Recent reports have generated an active debate in the scientific community on the active participation of SCs in the atrophic response, or whether muscle fiber alterations exclusively account for atrophy. In this manuscript, we review the experimental evidence supporting either view.

Several studies have investigated the effects of atrophic stimuli on muscle fibers and have identified key signaling pathways operating within the fiber that contribute to a decrease in fiber cross-sectional area. These studies highlighted the importance of protein homeostasis in regulating muscle mass, and identified the ubiquitin-proteasome and the autophagy-lysosome machineries as the two most important protolithic systems controlling protein turnover in muscle fibers. Several recent reviews describe the advances in understanding signaling mechanisms controlling the activity of these two systems in skeletal muscle fibers (Sandri, 2013; Schiaffino et al., 2013). In this review, we focus on the signaling pathways triggered in SCs in response to various atrophic stimuli. Moreover, we review the studies that address whether these molecular changes can be suppressed or reversed to induce SCs to increase the mass of atrophied muscles.

## Does SC Activity Affect the Loss of Muscle Mass?

Muscle homeostasis depends on a fine balance between catabolism and anabolism. A large body of evidence indicates that alterations in the control of protein turnover modify this balance and play an important role in essentially all forms of muscle atrophy (Sandri, 2008). Nevertheless, the mass of every tissue, including skeletal muscle is not only dependent on protein turnover, but also on cellular turnover (Sartorelli and Fulco, 2004). In keeping with this, muscle fibers are lost in certain forms of muscle wasting (Lexell et al., 1988). Moreover, muscle fibers are multinucleated structures, and it has been proposed that there are distinct “myonuclear domains”, whereby each myonucleus governs the surrounding cytoplasm by producing enough protein to support only a limited portion of the fiber (Mitchell and Pavlath, 2004). Although some studies report a reduction in muscle fiber size without a change in myonuclear number (Wada et al., 2002; Gundersen and Bruusgaard, 2008), several reports have documented a decrease in the number of myonuclei accompanying different forms of atrophy, supporting the hypothesis that myonuclear turnover and a reduction in muscle mass are causally related (Darr and Schultz, 1989; Schmalbruch et al., 1991; Allen et al., 1995, 1996; Day et al., 1995; Hikida et al., 1997). More importantly, this raises the prospect that alterations in myonuclear turnover can stabilize the reduction in muscle mass, at least under certain atrophic conditions.

As myonuclei are postmitotic, the need to replenish lost myonuclei to maintain a constant myonuclear number must come from myogenic progenitors that are able to fuse with the fibers. SCs, which have been reported to contribute to a large fraction of the fiber myonuclei during regeneration and growth, represent a natural candidate for this role (Moss and Leblond, 1971). In keeping with this idea, ablation of SCs in a paired box protein (Pax7)^DTR^ knock-in mouse model revealed a 20–40% loss of muscle mass 2 weeks after intramuscular injection of diphtheria toxin, an effect that persisted for 7 weeks after SC elimination and was exacerbated with strenuous resistance exercise (Sambasivan et al., 2011). Another study demonstrating an essential role for miRNAs in SC quiescence observed mild muscle atrophy within 6 months in uninjured mice that expressed a SC-specific conditional knockout (KO) of the gene *Dicer* (Cheung et al., 2012). These reports raise the tantalizing possibility that despite a low turnover rate in healthy muscle (Spalding et al., 2005), SCs could have an impact on homeostatic control of muscle mass. In contrast, a study using a genetic approach that allows for long-term depletion of SCs upon tamoxifen intraperitoneal injection in sedentary mice challenges this view (Fry et al., 2015). Despite the low regenerative capacity, these mice do not present signs of atrophy in hind limb muscles 1 month after tamoxifen administration and do not display exacerbated atrophy in 2-year-old mice. Therefore, these findings suggest that skeletal muscles do not necessarily require stem cell participation for tissue maintenance, at least under non-stressful conditions (Fry et al., 2015). Intriguingly, in a recent study, SCs were genetically labeled in adult mice and their fusion to myofibers in the absence of injury was followed throughout the lifespan of the mice. These experiments showed a contribution of SCs to myofibers in all muscles considered, although the extent and timing of their involvement differed in distinct muscles (Keefe et al., 2015). Importantly, the ablation of SCs using a genetic approach similar to that used by Fry et al. (2015) also showed a muscle group-specific response. In corroboration with the Fry et al. (2015) data, limb muscles were not significantly affected 6 months after SC depletion (Keefe et al., 2015). However, the diaphragm and extra ocular muscles displayed smaller fibers after 6 months of depletion of SCs, a decrease that was not exacerbated at later time-points (Keefe et al., 2015). Although a possible explanation for the different results derived from the individual studies could depend on the different nature of the animal models and experimental settings employed, the different types of muscles analyzed, or on the potential stress derived from the intramuscular injection of diphtheria toxin (Sambasivan et al., 2011), further investigations appears to be necessary to clarify the role of SCs in muscle homeostasis.

The discussion on the role played by SCs in the homeostatic maintenance of mass in healthy muscles has broader implications for the study of SC involvement in counteracting muscle mass loss under atrophying stimuli. The long-term SC depletion study conducted by Fry and colleagues, whereby the absence of SCs did not exacerbate sarcopenia in 2-year-old mice, is suggestive of the notion that SCs do not exert a compensatory action to counteract atrophy with age (Fry et al., 2015). Despite the compelling observations made in this study, the conclusions could possibly be limited by the incomplete depletion of SCs obtained in aged mice (an average of ~83%; Fry et al., 2015). Notably, elimination of ~85% of SCs by freeze- or cardiotoxin-mediated injury still results in muscle regeneration (Gayraud-Morel et al., 2007). Therefore, it is particularly relevant that the study from Keefe and colleagues using a mouse strain which allowed for the ablation of >95% of the SCs, obtained results that were similar to those reported by Fry and colleagues. When mice depleted of SCs were analyzed at 20 months of age, the contribution of SCs to myofiber maintenance appeared to be minimal in most hind limb muscles, with the striking exception of extensor digitorum longus (EDL) muscles that displayed a ~15% decline in fiber size (Keefe et al., 2015). Although an age-related reduction in fiber cross-sectional area is apparent at 20 months of age in most muscles, it is mainly after 2 years that a dramatic loss of muscle mass occurs in mice, which correlates with the appearance of an irreversible pre-senescent state in SCs that prevents activation and expansion (Sousa-Victor et al., 2014). The appearance of severe sarcopenia in older mice opens up the possibility for a primary requirement for SC contribution at later stages. Therefore, it would be interesting to extend the ablation studies to mice older than 2 years of age.

Intriguingly, several reports indirectly suggest that SCs may play a role in ameliorating sarcopenia, and that sarcopenia could be at least in part, a consequence of defective SC function. Several changes affecting the SC compartment have been observed in aged muscles (Alway et al., 2014). Strikingly, stem cell function and consequently regenerative potential are severely affected by aging in different tissues, including skeletal muscle (Liu and Rando, 2011). Alterations in the muscle and systemic environment occur during the aging process, thereby contributing to reversible and irreversible changes in SCs (Brack and Rando, 2007; Jang et al., 2011; Sousa-Victor et al., 2015). Moreover, despite the lack of a clear consensus, a reduction in SC number has been reported during aging, and it has been correlated with a reduction in fiber myonuclear content (Brack et al., 2005). Presence of centrally-nucleated fibers and an up-regulation of Myogenic differentiation (MyoD), Myogenin and embryonic Myosin Heavy Chain expression, which are generally considered as hallmarks of fiber regeneration, have also been documented in aging muscle (Edström and Ulfhake, 2005). Notably, a loss of myonuclei with aging in *MyoD* KO mice was exacerbated compared to wild-type mice. Since SCs display defective differentiation in the absence of MyoD expression, this observation has been interpreted as evidence in favor of a role for SCs in replenishing fiber myonuclei during aging (Brack et al., 2005).

An up-regulation of MyoD expression has also been documented in other forms of atrophy, and is particularly well established in denervation models of atrophy (Legerlotz and Smith, 2008). Upon denervation, SCs proliferate transiently, but do not progress through differentiation and form small immature (embryonic Myosin Heavy Chain^+ve^) fibers, indicative of an unsuccessful attempt to restore muscle mass and function (Viguie et al., 1997; Borisov et al., 2005; Doppler et al., 2008). As in aging muscle, the increase in MyoD expression observed in denervated muscle could be interpreted as a result of SC activity. Nevertheless, it is notable that muscle fibers also express MyoD at low levels, even in the absence of ongoing regeneration (Hughes et al., 1993). It is therefore possible that the increase in MyoD expression in muscle observed after denervation, and possibly in other models of atrophy as well, could constitute a SC-independent response of the fibers to atrophic stimuli (Koishi et al., 1995). Indeed it has been proposed that the induction of MyoD could represent an attempt by muscle fibers to regain sensitivity to neural activity. In keeping with this idea, MyoD has been shown to regulate the expression of Acetylcholine receptor (AChR), the expression of which is also increased upon denervation (Legerlotz and Smith, 2008). On similar lines, Myogenin is also expressed in fibers in the absence of a regenerative response (Hughes et al., 1993), and can act as an essential mediator of neurogenic atrophy by regulating the expression of Murf1 and Atrogin-1 within the fiber, thereby promoting muscle proteolysis (Moresi et al., 2010).

Alterations in the myogenic program have also been described in cachexia, a complex metabolic syndrome characterized by a loss of muscle mass, which is initiated by underlying illnesses of different nature, such as cancer, CHF, COPD, CKD, burns, chronic infection and sepsis (Evans et al., 2008; Fearon et al., 2012). Changes in the expression of myogenic factors and impaired differentiation have been reported in cachectic muscles (Coletti et al., 2005; Langen et al., 2006; Schwarzkopf et al., 2006; Penna et al., 2010; Zhang et al., 2010a; Wu et al., 2013). In tumor-bearing mice that recapitulated clinical features of cancer-induced cachexia, as well as in muscle biopsies from patients with pancreatic cancer, a significant expansion of cells expressing high levels of the SC-marker Pax7 was observed (Penna et al., 2010; He et al., 2013). Intriguingly, the majority of the Pax7^+ve^ cells were noted in the interstitium and a fraction of them were reported to express mesenchymal (Pdgfr*-α*, Sca1) and pericyte (NG2) markers, which are not expressed in the SC lineage (He et al., 2013). These data reveal that cancer-induced atrophy triggers myogenic commitment in multiple stem cell progenitors, in addition to the SC population (He et al., 2013). In stark contrast with other atrophy models (see above), MyoD and Myogenin expression are not significantly induced in tumor-bearing mice compared to controls, and an impaired myogenic program prevents these cells from efficiently fusing with existing myofibers and therefore ameliorating the wasting condition (He et al., 2013). Pax7 has been reported to inhibit differentiation by suppressing MyoD and Myogenin expression (Olguin and Olwin, 2004). Positive or negative modulation of Pax7 expression in cachectic mice impact muscle mass in a negative or positive manner, respectively. Moreover, depletion of Pax7^+ve^ cells further exacerbated muscle loss, directly indicating that stem cells are able to counteract muscle atrophy in cachectic mice. Nevertheless, the persistent expression of Pax7 stalls the myogenic program and limits the functionality of muscle stem cells (He et al., 2013). In line with the idea that SC functionality can compensate for the effects of atrophic stimuli, *mdx* mouse models of Duchenne muscular dystrophy (DMD) showed a correlation between muscle mass and alterations in regenerative potential. Specifically, during early stages of pathology characterized by effective regeneration, a compensatory hypertrophy has been observed. However, at later stages when regeneration becomes defective, there is a dramatic decrease in muscle mass (Mouisel et al., 2010).

Alterations in SC function, including defective fusion properties, have also been observed in the hind limb suspension model of disuse atrophy (Mitchell and Pavlath, 2004). Conversely, changes in SC numbers are not a consistent finding in disuse atrophy, reflecting the complexity of events occurring in this context (Brooks and Myburgh, 2014). Notably, the decrease in muscle mass accompanying different pathophysiological conditions displays variable changes in SC numbers depending on the type and the severity of the atrophic stimulus (Table 1). Also the time-scale of the analysis appears to play an important role in SC quantification, as highlighted in denervation experiments, in which an initial phase of proliferation is followed by a decrease in numbers of SCs (Viguie et al., 1997). The inconsistent reports on changes in SC numbers, together with the observations that alterations in SC function appear to be a common feature in most atrophic conditions, raises the prospect that rather than absolute numbers, it is the functionality of the SCs that is more relevant in their ability to counteract atrophy. Further studies will be required to confirm this view, and to conclusively impart a non-redundant role for SCs and other muscle resident stem cells in specific atrophic processes.

**Table 1:** **Satellite cell alterations in different atrophic conditions**.

Condition	Changes in number	Changes in function	Notes	References
Aging	↓ =	Yes	↓ proliferation ↓ myogenic differentiation	Snijders et al. (2009) and Alway et al. (2014)
Cachexia	↑	Yes	↑ activation/proliferation ↓ differentiation/fusion	Penna et al. (2010) and He et al. (2013)
Denervation	↑*	Yes	↑ activation/proliferation ↓ fusion	Viguie et al. (1997), Borisov et al. (2005) and Doppler et al. (2008)
Hind limb suspension	↓ = ↑	Yes	↓ proliferation ↓ differentiation	Mitchell and Pavlath (2004) Brooks and Myburgh (2014)
Glucocorticoids	n.d.	Yes	↓ proliferation ↓ differentiation	Dong et al. (2013)
DMD	↑	Yes	↓ proliferation ↓ myogenic differentiation/fusion	Blau et al. (1983) and Delaporte et al. (1984) Jasmin et al. (1984), Melone et al. (1999) and Biressi et al. (2014)

↑, Increase; ↓, decrease; =, unchanged; n.d., not defined; * initial increase, but reduced in the long-term (Viguie et al., 1997).

## Molecular Alterations in SCs During Atrophy

**Figure 1:**
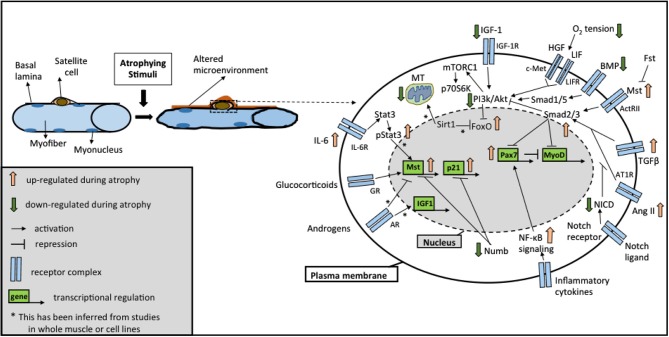
**Overview of the signaling pathways in satellite cells (SCs) during atrophy**. Schematic representation of the major signaling pathways implicated in SC biology under different atrophic stimuli (see text for details). Cross-talks between different pathways are highlighted by positive and negative regulation at different levels. Abbreviations: AR, androgen receptor; GR, glucocorticoid receptor; Il-6R, Interleukin-6 receptor; IGF-1R, IGF-1 receptor; LIFR, LIF receptor; AT1R, Angiotensin II receptor 1; ActRII, Activin receptor 2; Fst, Follistatin; Mst, Myostatin; NICD, Notch intracellular domain; MT, mitochondria.

It is increasingly becoming clear that distinct signaling pathways mediate a common outcome of a loss of muscle mass observed in instances of atrophy, sarcopenia, or cachexia (Glass, 2005; Fanzani et al., 2012). While the involvement of these molecular mechanisms has been described in great detail in myofibers in response to atrophic stimuli (Bonaldo and Sandri, 2013; Schiaffino et al., 2013), in this section we review alterations in these pathways occurring in SCs accompanying fiber atrophy. Moreover, we focus on studies reporting functional changes in SCs resulting from alterations in these pathways, which possibly contribute to the loss of muscle mass. Importantly, these pathways extensively modulate one another and coordinate overlapping responses not only in the muscle fiber, but also in the SC compartment (Figure 1). Below, we discuss five major signaling pathways traditionally associated with skeletal muscle atrophy: (a) insulin-like growth factor (IGF)-Akt-FoxO signaling; (b) Transforming Growth Factor Beta (TGFβ) superfamily signaling; (c) Glucocorticoids and androgen signaling; (d) nuclear factor kappa-light-chain-enhancer of activated B cells (NF-κB) signaling; (e) Sirtuin 1 (Sirt1) signaling and mitochondrial dysfunction. Additionally, we review recent studies involving (f) Notch signaling, a pathway well known to influence SC functionality for a role in regulating muscle mass.

### IGF-Akt-FoxO Signaling

A central pathway regulating fiber size is the IGF-Akt pathway (Rommel et al., 2001). Activation of this pathway triggers the activation of mammalian target of Rapamycin (mTOR) that results in the phosphorylation of its targets p70 S6K and 4E-BP1 (Bodine et al., 2001; Pallafacchina et al., 2002; Wullschleger et al., 2006). An additional consequence of these growth- promoting stimuli is the inhibition of the FoxO family of transcription factors that are key in initiating the atrophy program (Sandri et al., 2004). Consistent with this finding, components of the FoxO pathway were found to be increased in skeletal muscle during sepsis, cancer cachexia, and treatment following lipopolysaccharide (LPS), glucocorticoid, and cytokines (Liu et al., 2007; Schmitt et al., 2007; Crossland et al., 2008; Moylan et al., 2008). Additionally, the ability of the FoxO transcription factors to regulate muscle mass during disuse atrophy and following glucocorticoid treatment via the regulation of atrophy-related genes has been well documented (Kamei et al., 2004; Lecker et al., 2004; Sandri et al., 2004; Senf et al., 2010).

Only recently have studies begun to address the influence of IGF1 signaling in the behavior of SCs accompanying the atrophic response in fibers. For instance, in CKD-induced muscle atrophy, SCs displayed a reduction in phosphorylated Akt levels indicative of impaired IGF-1 signaling, and a decrease in activation and myogenic progression (Zhang et al., 2010a). Moreover, both CKD and *IGF-1 receptor* KO mice developed fibrosis in regenerating muscles, suggesting a decline in SC functionality (Zhang et al., 2010a). Similarly, old dystrophic *mdx* mice also displayed reduced Akt phosphorylation along with defective regeneration and atrophy (Mouisel et al., 2010). In studies aimed at addressing the role of the FoxO’s in SCs, overexpression of FoxO3 in myogenic progenitors decreased their proliferation (Rathbone et al., 2008). Similarly, abolishing FoxO activity either by injecting dominant negative (DN) FoxO-expressing plasmid into murine muscles or deleting the *Foxo3* gene specifically in SCs, resulted in an increase in the proliferation of SCs (Reed et al., 2012; Gopinath et al., 2014). Interestingly, the former study reported a hypertrophic response following global suppression of FoxO activity, which acts as a protective mechanism to suppress atrophy following sepsis and cancer (Reed et al., 2012). However, while FoxO functionality may support atrophy when expressed in the fiber, the expression of a specific isoform, FoxO3, in SCs seems to be a prerequisite for maintaining the regenerative capacity of muscle and might therefore act to protect the muscle from atrophying stimuli (Gopinath et al., 2014). These paradoxical observations suggest that FoxO activity promotes different programs in SCs vs. fibers, and underscores the importance of identifying the biochemical processes that promote atrophy in combination with a SC-focused approach.

### TGFβ Superfamily Signaling

Members of the TGFβ superfamily are potent regulators of muscle mass (Goodman and Hornberger, 2014). Canonical TGFβ signaling operates through the Smad signaling cascade. Specifically, the TGF-βs, Activins, and some members of the growth differentiation factor (GDF) subfamilies activate a Smad2/3-dependent signaling cascade, whereas members of the bone morphogenetic protein (BMP) subfamily primarily induce a Smad1/5/8-dependent signaling mechanism (Massagué, 1998). An increasing amount of evidence has implicated Smad2/3 in possessing a catabolic function, while Smads 1/5/8 have an anabolic function (Goodman and Hornberger, 2014). Members of the TGFβ superfamily play an important role in controlling the proliferation and differentiation of myogenic cells, suggesting that the effects exerted on muscle mass are in part achieved by controlling SC function (Kollias and McDermott, 2008).

Myostatin (GDF8), is a member of the TGFβ superfamily and a potent inhibitor of muscle growth, such that various mammalian species bearing *Myostatin-null* mutations display a hypertrophic response (Kambadur et al., 1997; Lee and McPherron, 2001; Schuelke et al., 2004). Myostatin binding to Activin receptor 2 (ActRII) and Activin receptor-like kinase 4 and 5 (ALK 4/5) activates the Smad2/3-dependent signaling cascade and blocks the Akt-mTOR growth- promoting pathway (Rommel et al., 2001). Systemic administration of Myostatin in adult mice was found to induce profound muscle loss (Zimmers et al., 2002). Moreover, enhanced levels of Myostatin have been observed in a variety of muscle wasting conditions (Gonzalez-Cadavid et al., 1998; Lalani et al., 2000; Yarasheski et al., 2002; McKay et al., 2012). Importantly, Myostatin was suggested to play a role in negatively regulating SC activity by inhibiting myogenic progression (Langley et al., 2002; McCroskery et al., 2003; McFarlane et al., 2008; Trendelenburg et al., 2009). Consistent with the notion that modulation of Myostatin expression is bound to have a significant impact on muscle mass and SC behavior, it was found that inhibition of the ActRIIB pathway stimulated SC proliferative potential and reversed muscle wasting in cancer-bearing mice (Zhou et al., 2010). Moreover, altered levels of Myostatin and its receptor resulted in impaired SC proliferation and differentiation in muscle wasting accompanying liver cirrhosis in a portacaval anastomosis (PCA) rat model (Dasarathy et al., 2004). While it has been suggested that a loss in body weight of the PCA rats compared to sham controls is an overall consequence of specific fiber type atrophy (type 2) and impaired SC functionality, what remains to be determined is a quantitative assessment of SC dysfunction alone that contributed to the atrophy observed in this model.

Several studies have provided insights into mechanisms operating upstream and downstream of Myostatin, thereby identifying diverse signaling pathways that have a shared outcome of a loss of muscle mass. Indeed, it has been shown that Numb, a mediator of asymmetric cell division suppresses Myostatin expression, such that *Numb-*deficient SCs display impaired proliferation characterized by high levels of p21 and Myostatin (George et al., 2013). A decrease in muscle mass was apparent after developmental deletion of *Numb* (George et al., 2013). Given these results, it would then be interesting to investigate whether SC-specific *Numb* conditional KO animals display a reduction in muscle mass, and if the loss is exacerbated in the background of liver cirrhosis, cancer cachexia or other muscle wasting conditions. Intriguingly, lower levels of Numb expression have been reported in muscle biopsies from older men (60–75 years old) than in muscles from younger men (18–25 years old; Carey et al., 2007).

Recent reports have implicated a role for Stat3, a downstream effector of Interleukin 6 (IL-6) in muscle wasting (Muñoz-Cánoves et al., 2013; Zhang et al., 2013). IL-6, an inflammatory cytokine is itself known to be involved in initiating muscle wasting when present systemically and for sustained periods of time (Strassmann et al., 1992; Haddad et al., 2005). Elevated levels of activated Stat3 (p-Stat3) were shown to initiate Myostatin-mediated muscle wasting and inflammation in patients with CKD or diabetes via increase in C/EBP δ levels (Zhang et al., 2013). Consistent with this, muscle-specific deletion of *Stat3* and inhibition of CCAAT/enhancer-binding protein gamma (C/EBP δ) expression countered the loss of muscle mass in CKD (Zhang et al., 2013). While this study did not address the status of the Stat3 pathway and the consequences of its modulation within SCs, another study demonstrated that conditional ablation of Stat3 specifically in SCs increased their expansion during regeneration, but compromised their myogenic differentiation and prevented their contribution to regenerating myofibers (Tierney et al., 2014). Intriguingly, transient inhibition of Stat3 function by pharmacological treatment led to an expansion of SCs at a higher rate, and maintained their ability to differentiate into fibers, thus enhancing tissue repair in both aged and dystrophic muscle (Price et al., 2014; Tierney et al., 2014).

Recent findings have proposed a key role for BMPs in controlling muscle mass. Unlike Myostatin signaling, BMP acts through Smad1/5/8, eliciting a hypertrophic response in muscle and involving the Akt pathway, the inhibition of which by Rapamycin attenuates at least partially the BMP-mediated response (Sartori et al., 2013; Winbanks et al., 2013). Inhibition of BMP signaling causes muscle atrophy, abolishes the hypertrophic phenotype of *Myostatin-null* mice, and exacerbates the effects of denervation and fasting (Sartori et al., 2013; Winbanks et al., 2013). The atrophic response observed after fiber-specific *Smad4* conditional ablation suggests that BMPs regulate muscle mass by directly acting on muscle fibers (Sartori et al., 2013). Indeed, the degradation of muscle fiber proteins stimulated by the ubiquitin ligase MUSA1 has been implicated in this process (Sartori et al., 2013; Winbanks et al., 2013). Importantly, the inhibition of BMP signaling not only counteracts the increase in muscle mass in *Myostatin-null* mice, but also blunts the hypertrophic response induced by Follistatin (Winbanks et al., 2013). Follistatin is a powerful regulator of muscle mass, which exerts its function mainly by inhibiting the action of Myostatin and Activins, both of which are implicated as negative regulators of muscle growth (Link and Nishi, 1997; Souza et al., 2008; Gilson et al., 2009; Lee et al., 2010). Strikingly, the presence of SCs seems to be required for a full Follistatin-dependent hypertrophy, as muscle irradiation which abolishes the proliferative capacity of SCs blunted the effects of Follistatin overexpression on muscle mass (Gilson et al., 2009). A caveat in this set of experiments is that the use of electroporation to introduce Follistatin-expressing plasmids into muscles is accompanied by a regenerative response that almost certainly involves SC participation (Skuk et al., 2013). Nevertheless, since Follistatin-induced hypertrophy is mitigated by both the inhibition of BMP signaling as well as by blocking cell proliferation, it is conceivable that BMP-mediated hypertrophy is dependent on SC activity to a significant extent. Indeed, BMP signaling strongly inhibits the myogenic differentiation program in myogenic precursor cells, and is able to induce features typical of the osteoblast lineage in myogenic cell lines under specific *in vitro* conditions (Murray et al., 1993; Katagiri et al., 1994). While there is a lack of evidence of the ability of SCs to commit to the osteogenic program *in vivo*, several observations indicate that BMP signaling plays an important role during muscle regeneration by controlling myogenic progenitor differentiation and positively modulating their proliferative expansion (Lounev et al., 2009; Clever et al., 2010; Friedrichs et al., 2011; Ono et al., 2011). Interfering with normal BMP signaling *in vivo* leads to smaller regenerated fibers after muscle injury and to smaller muscles during fetal development (Wang et al., 2010; Ono et al., 2011). Together, these observations suggest that BMP signaling occupies a central position in a complex network of signals that control SC biology and muscle mass, besides being implicated in the etiology of atrophy. These observations also support the idea of an active cross-talk between different branches of the TGFβ signaling pathway in the control of muscle mass.

An involvement of SCs in the control of muscle mass is particularly relevant in diseases that continually demand a damage-repair response from the tissue, such as muscular dystrophies. Muscular dystrophies form a group of heterogeneous genetic diseases, often characterized by progressive muscle atrophy (Shin et al., 2013). Little is known about the molecular changes occurring in SCs within the dystrophic environment that prevents them from restoring the growth of the atrophied muscles. Several members of the TGFβ superfamily, including Myostatin, TGFβ1, and TGFβ2 have been implicated in the progression of different forms of muscular dystrophy (Yamazaki et al., 1994; Bogdanovich et al., 2002; Wagner et al., 2002; Andreetta et al., 2006; Onofre-Oliveira et al., 2012; Biressi et al., 2014). Enhanced TGFβ signaling appears to be responsible for the aberrant myogenic program observed in the muscles of *mdx* mice (Biressi et al., 2014). TGFβ signaling appears to alter SC function also in other diseases, such as Emery-Dreyfuss muscular dystrophy (AD-EDMD), in which mutations in the *Lamin A/C* genes cause muscle atrophy and necrosis (Sewry et al., 2001). An analysis of SC activity in the *Lmna*^−/−^ mice revealed that the proliferation rates and kinetics of activation of the SC progeny derived from *Lmna*^−/−^ muscles were slower and delayed compared to wild type muscles (Cohen et al., 2013). More importantly, *Lmna*^−/−^ myoblasts displayed elevated levels of Smad2/3 that did not localize to the nucleus in response to TGFβ stimulation. As a result, there was an increase in cell death in myotube cultures, an event that could contribute to wasting (Cohen et al., 2013). Additionally, defective SC behavior, fiber atrophy, and enhanced TGFβ signaling have also been observed in mouse models of *Caveolin 3*-deficient limb-girdle muscular dystrophy (Ohsawa et al., 2012). Importantly, administration of an inhibitor of TGFβ type I receptor not only ameliorates muscle atrophy, but also restores the number and differentiation potential of SCs, indicating that both TGFβ-dependent reduction in SCs and impaired myoblast differentiation contribute to the cellular mechanism underlying *Caveolin 3*-deficient muscle atrophy (Ohsawa et al., 2012).

A key modulator of TGFβ signaling in muscle and an effector molecule of the renin-angiotensin system, Angiotensin II (Ang II), has been implicated in muscle degeneration and accumulation of fibrotic tissue in several congenital and acquired muscle disorders (Cohn et al., 2007). In keeping with this, down-regulation of TGFβ signaling by Ang II inhibitors ameliorates muscle wasting in different myopathies (MacDonald and Cohn, 2012). Ang II is also involved in the etiology of cachexia, such that patients with CKD and CHF often have elevated Ang II levels, and treatment with an angiotensin converting enzyme inhibitor can reduce weight loss (Anker et al., 2003). Consistent with this notion, earlier studies showed that the infusion of Ang II in rodents decreased muscle weights by increasing protein degradation, disrupting IGF-1 signaling and inducing apoptosis (Brink et al., 1996). In SCs, Ang II was demonstrated to impair SC proliferation and differentiation by signaling events mediated by one of its receptors, Angiotensin 1 (AT1R), during the course of muscle regeneration (Yoshida et al., 2013). Indeed, by inhibiting AT1R activity in CHF, a condition in which high Ang II levels are present, the observed reduction in SC numbers and muscle weight could be blocked (Yoshida et al., 2013). Intriguingly, an opposite function was ascribed to Ang II Type 2 receptor (AT2R) that is expressed in differentiating SC progeny and is known to promote regeneration (Yoshida et al., 2014). These observations highlight the importance of defining the mechanisms operating downstream of atrophic signals, in order to develop therapeutic approaches that are specific and effective.

### Glucocorticoid and Androgen Signaling

Glucocorticoids, either in their synthetic or physiological form, cortisol (in human) and corticosterone (in rodents), have emerged as potent negative regulators of muscle mass (Braun and Marks, 2015). Elevated levels of circulating glucocorticoids have been linked to different atrophic conditions including sepsis, diabetes, and cancer (Braun and Marks, 2015). Glucocorticoids act by binding to glucocorticoid receptors, a family of nuclear receptors, and exerting their function using different mechanisms, including stimulating the expression of muscle-specific E3 ubiquitin ligases in muscle fibers and modulating the function of Akt, Myostatin, and multiple other signaling pathways involved in the control of muscle mass (Braun and Marks, 2015). By stimulating Myostatin expression, glucocorticoids can also decrease SC proliferation and differentiation (Dong et al., 2013).

Importantly, the effects of glucocorticoid administration on muscle mass are counteracted by androgens in both patients and animal models (Creutzberg et al., 2003; Eason et al., 2003). Androgens are efficient positive modulators of muscle mass and function by binding to the androgen receptor, also a member of the nuclear receptor superfamily (Dubois et al., 2012). The androgen receptor is expressed not only in muscle fibers, but also in SCs, in particular in the *levator ani* muscle, a muscle that is dramatically affected in animal models with a conditional ablation of androgen receptor expression in SCs and fibers (Swift-Gallant and Monks, 2013; Dubois et al., 2014). Several reports suggest that androgens are able to modulate gene expression and function in SCs and myoblast cell lines (Chen et al., 2005). Testosterone, the principal circulating androgen is decreased in the serum of older men, possibly contributing to the SC dysfunction observed in this condition (Yialamas and Hayes, 2003). Intriguingly, the activation of SCs observed in denervated *levator ani* muscle does not occur in castrated rats, and this correlates with a reduction in myonuclear number accompanying the atrophy observed in denervated muscles of castrated rats (Nnodim, 1999). These observations suggest that at least in specific muscles and under specific atrophic conditions, the control of SC activity by androgens may contribute to fiber homeostasis.

### NF-κB Signaling

A large body of evidence has implicated the NF-κB transcription factors as being major modulators of muscle mass (Li et al., 2008; Peterson et al., 2011). When inactive, NF-κB is maintained in the cytoplasm by a class of proteins called IκB. In response to inflammatory cytokines, such as TNFα, IκB is degraded leading to the nuclear translocation of NF-κB and activation of NF-κB-dependent transcription (Li et al., 2008; Peterson et al., 2011). An increase in NF-κB signaling has been reported in the atrophic response associated with different disease conditions. NF-κB signaling has been shown to mediate atrophy by increasing the expression of components of the ubiquitin-proteasome system, and by promoting the atrophic effects of inflammation-related proteins (Li et al., 2008; Peterson et al., 2011). More importantly, an increasing number of observations suggest that NF-κB also regulates SC activity in different atrophic conditions. Specifically, the presence of systemic inflammation accompanying clinical features associated with cachexia led to the discovery of a cytokine-induced up-regulation of NF-κB signaling in the suppression of myogenic gene expression in cell culture (Guttridge et al., 2000). Moreover, a detailed examination of events within muscles from tumor-bearing mice revealed that the induction of NF-κB activity by cachectic serum contributed to Pax7 dysregulation in muscle-resident progenitors, and was followed by a significant decline in muscle mass (He et al., 2013). Although these observations do not distinguish between increases in NF-κB levels in SCs vs. fibers, there is a clear indication that together with functioning as a potent modulator of muscle regeneration and myogenic differentiation, NF-κB signaling promotes atrophy, an effect which is achieved in part by altering the behavior of SCs (Langen et al., 2001; Dogra et al., 2006; Mourkioti et al., 2006; Wang et al., 2007; Bakkar et al., 2008).

### Sirt1 and Mitochondrial Dysfunction

Mitochondrial function and metabolism is crucial for SC activation, proliferation, and for efficient muscle regeneration (Jash and Adhya, 2012; Rodgers et al., 2014). A reduction in mitochondrial mass, increased damage to mitochondrial DNA, and increased levels of reactive oxygen species (ROS) produced by the existing mitochondria were observed with age (Minet and Gaster, 2012; Wang et al., 2013). Sirt1 is a potent regulator of mitochondrial metabolism, displaying altered expression in tumor-bearing animals and contributing to reduced regeneration in muscle wasting (Toledo et al., 2011). Intriguingly, an increase in Sirt1function is one of the many outcomes of caloric restriction, that also include an increase in SC proliferation, an increase in mitochondrial abundance and an enhancement of the regenerative capacities of muscles (Lee et al., 1998; Cohen et al., 2004; Cerletti et al., 2012; McKiernan et al., 2012). These observations support a possible role for Sirt1 in reducing muscle loss that occurs with aging. Indeed, Sirt1 overexpression has been reported to block fasting and denervation-induced fiber atrophy by reducing FoxO activity (Lee and Goldberg, 2013). Moreover, Sirt1 controls the transcription of peroxisome proliferator-activated receptor-gamma coactivator 1 alpha (PGC1α), which in turn induces mitochondrial biogenesis and regulates peroxisome proliferator-activated receptors gamma (PPARδ), a positive regulator of SC proliferation (Amat et al., 2009; Angione et al., 2011). Consistent with this observation, over-expression of Sirt1 increases the proliferation of myogenic progenitors (Rathbone et al., 2009). Moreover, as a consequence of ablating Sirt1 in SCs, myogenic progenitors undergo premature differentiation, thereby negatively affecting muscle regeneration and growth (Ryall et al., 2015). Although additional mechanistic studies are required, this body of evidence suggests that the Sirt1-PGC1α axis could ameliorate the loss of muscle mass at least in part by improving mitochondrial function and the regenerative potential of SCs.

### Notch Signaling

The importance of Notch signaling during muscle development and regeneration has been well established (Luo et al., 2005; Vasyutina et al., 2007a; Mourikis and Tajbakhsh, 2014). Notch signaling plays an important role in maintaining quiescence, proliferation, homing and self-renewal capabilities of SCs (Conboy and Rando, 2002; Bjornson et al., 2012; Bröhl et al., 2012; Mourikis et al., 2012b; Wen et al., 2012; Gopinath et al., 2014). Notch signaling is essential to maintain muscle progenitors during fetal development, to promote their expansion, and to generate SCs (Vasyutina et al., 2007b; Mourikis et al., 2012a). Indeed, developmental inactivation of the Notch transcriptional complex by the selective ablation of the Notch regulators, recombination signal binding protein J (RBP-J) or mastermind-like (MAML1), specifically in the myogenic compartment resulted in the reduction of muscle mass (Vasyutina et al., 2007b; Lin et al., 2013).

Aging muscles consist of SCs with decreased regenerative potential, which in turn is an outcome of reduced Notch signaling, and can be restored by exposure to a young systemic environment (Conboy et al., 2003, 2005). Intriguingly, it was also reported that the consequences of Vitamin D depletion in aged rats exacerbated the muscle loss associated with aging and was accompanied by reduced Notch activity in these muscles (Domingues-Faria et al., 2014). At this point it remains to be established whether Vitamin D exerts its effects on skeletal muscle through the inhibition of the Notch signaling pathway. Additionally, it remains to be demonstrated whether enhancing Notch function in the Vitamin D-depleted aged rats display possible beneficial effects on muscle mass, in addition to the involvement of SCs in this context. Intriguingly, the observation that Notch activation was able to abrogate the inhibitory effects of the cachectic factor Ang ll on proliferation of myogenic progenitors in culture raises the possibility of using positive modulators of Notch signaling to boost SC proliferation and counteract muscle loss (Yoshida et al., 2013).

However, recent literature suggests that the outcome of manipulating Notch activity has been met with incongruous results. Using a Notch reporter mouse, it was demonstrated that SCs isolated from *mdx* mice displayed reduced activation of Notch signaling, and that constitutive Notch activation could at least in part rescue the self-renewal deficit observed in *mdx* SCs, without ameliorating muscle pathology associated with these dystrophic mice (Jiang et al., 2014). On the other hand, a recent study observed that acute manipulation of Notch signaling by the injection of activators or inhibitors did not affect muscle mass or maximal force in *mdx* mice, as well as mice that were double-deficient for *Utrophin* and *Dystrophin* (Church et al., 2014). These observations suggest that while SCs display a stage-specific requirement for Notch signaling, dystrophic muscles present a complex environment with SCs at different stages of progression and consequently exhibit different responses to Notch manipulation that may not contribute to a net increase in muscle weight. These studies underscore the importance of not only investigating specific signaling pathways in the context of specific forms of atrophy, but also addressing the efficacy of manipulating a particular signaling mechanism for therapeutic purposes.

## Reversal of Atrophy: A Role for SCs?

The primary objective of obtaining a comprehensive understanding of the mechanistic processes underlying atrophy in a manner that discerns molecular changes in SCs from those in fibers, is to address whether manipulating these signaling mechanisms can enhance the participation of SCs in restoring muscle mass. In this section, we review those studies in which SC activity has been modulated to expand their functionality in muscle wasting accompanying chronic diseases. Broadly, the focus of these interventions has been signaling pathways that modulate SC proliferation, differentiation, senescence and survival.

In muscle wasting accompanying COPD, patients experience decreased oxygen saturation level (hypoxemia) that can elicit hypoxic responses in tissues (Wüst and Degens, 2007). This includes an impairment in anabolic pathways, decrease in food intake by the induction of leptin, and muscle disuse that are directly responsible for a loss in muscle mass (Wüst and Degens, 2007). In studies aimed to increase the protein synthesis pathway in SCs under conditions of hypoxia-induced atrophy, a regimen of alternating treatments with hepatocyte growth factor (HGF) and leukemia inhibitory factor (LIF) not only increased SC proliferation, but also increased the cross sectional area of fibers and total muscle weight (Hauerslev et al., 2014). Corroborating these results in normoxic mice, this growth factor treatment promoted SC proliferation and increased the weight of the *tibialis anterior* muscle in mice deleted for Myostatin expression (Hauerslev et al., 2014). These results further highlight the potential of exploring Myostatin regulation in SCs for therapeutic purposes in combatting atrophy.

Strategies aimed at enhancing SC proliferation to increase their participation in muscle mass restoration have been particularly useful in limb-girdle muscular dystrophy type 1B (LGMD1B) and AD-EDMD forms of dystrophies. Under these conditions, increased levels of lamina-associated polypepetide alpha (Lap2α), a protein that interacts with Lamin A/C and phosphorylated Rb has been implicated in reducing SC proliferation (Mancini et al., 1994; Ozaki et al., 1994). Since the *Lap2α*^−/−^ cells are hyperproliferative due to a defect in cell cycle exit, the authors created a *Lmna*^−/−^ *Lap2α*^−/−^ double KO mice to enhance SC proliferation, thereby resulting in an increase in the fusion index and overall muscle size of the double KOs (Cohen et al., 2013). This study not only offers valuable insights into the etiology of laminopathies but also provides alternate strategies using SCs for therapeutic intervention.

Contrary to the atrophic conditions discussed above, the increase in SC numbers in the interstitium in cancer-induced cachexia prompted the use of mutant mouse models to inactivate Pax7 expression in tumor-bearing mice (He et al., 2013). Not only did this cause a reversal of wasting by promoting cell differentiation and fusion with injured fibers, but also demonstrated that impaired myogenic progression by sustained Pax7 expression was the primary cause for muscle wasting in these mice, and offered the attractive possibility of gene therapy approaches to modulate Pax7 expression (He et al., 2013). In particular, the identification of regulators that repress Pax7 expression to limit its window of action and allow for myogenic progression during the course of normal regeneration can be potentially exploited for therapeutic purposes. Indeed, the activation of the Polycomb Repressive Complex 2 (PRC2) by p38α kinase results in the formation of repressive chromatin on the *Pax7 locus*, thereby providing an additional interventional target that could be explored in a tumor-promoting milieu (Palacios et al., 2010).

In disuse atrophy, the focus of various interventional studies have been on regulators that enhance SC functionality and increase muscle growth, especially in the subsequent recovery phase. In a recent study, E3 ubiquitin ligase tripartite motif-containing 32 (TRIM32) was demonstrated to be essential for the selective regrowth of Type 2 fast fibers after hind limb suspension-induced atrophy (Kudryashova et al., 2012). *TRIM32*-deficient myoblasts displayed impaired differentiation, and elevated levels of senescence-associated β-galactosidase (β-gal; Kudryashova et al., 2012). Premature senescence of SCs was also demonstrated to be the underlying cause for the pathogenic features associated with limb-girdle muscular dystrophy 2H (LGMD2H), that arise from mutations in *TRIM32* (Saccone et al., 2008; Cossée et al., 2009). These studies suggested that unlike other E3 ubiquitin ligases that promote atrophy, TRIM32 might possess a unique function of preventing premature senescence in SCs, thereby enhancing muscle growth. Although these speculations support the idea of an involvement of SCs, more investigations are required to explore the mechanisms by which TRIM32 could promote regrowth after atrophy. Indeed, the extent to which SCs are required in the process of recovery from hind limb suspension is still unclear. Ablation studies indicate that muscle mass recovery after hind limb suspension could also occur in the absence of SCs (Jackson et al., 2012). Nevertheless, during the recovery phase of soleus muscle mass upon its reloading, the decrease in myonuclear content derived from hind limb unloading was restored to control levels, suggesting that myogenic precursor cells can proliferate and fuse with myofibers during the recovery process (Mitchell and Pavlath, 2001). Moreover, after an initial phase of muscle regrowth, inhibiting the proliferation of muscle precursor cells by irradiation prevented a full recovery (Mitchell and Pavlath, 2001). As such, it would be crucial to quantify the relative contributions of SC-mediated and myofiber-mediated processes, in addition to investigating molecular events that distinguish between SC-intrinsic and fiber-intrinsic processes during muscle recovery.

IGF-1 is another factor that has been shown to modulate SC activity and has been reported to be beneficial in promoting regrowth after muscle unloading. In a study aimed to investigate the effects of IGF-1 overexpression on the recovery of muscle size during ambulation after cast immobilization, it was observed that viral-mediated IGF-1 transfer to skeletal muscle enhanced regeneration (Stevens-Lapsley et al., 2010). Intriguingly, IGF-1 overexpression did not protect against cast immobilization-induced muscle atrophy, indicating that there are different mechanisms regulating muscle mass during unloading and reloading (Stevens-Lapsley et al., 2010). Delivery of IGF-1 into muscle and muscle-specific overexpression of IGF-1 were also beneficial in ameliorating sarcopenia and stimulating recovery in immobilized old muscles (Barton-Davis et al., 1998; Chakravarthy et al., 2000; Musarò et al., 2001). Moreover, increased levels of IGF-1 in muscle has been shown to control SC activity and reduce muscle wasting in different genetic disorders, including muscular dystrophy and amyotrophic lateral sclerosis (ALS; Barberi et al., 2009).

A crucial aspect underlying the enhancement of SC functionality for cellular therapy is the investigation of mechanisms that specifically promote cell survival without affecting proliferation, in order to avoid the risk of cancer-promoting effects. A growth factor-derived engineered protein, Magic-Factor 1 (or Met-Activating Genetically Improved Chimeric Factor 1), has been developed to elicit the activation of the Akt survival pathway, but not the mitogenic ERK pathway. Magic-Factor 1 decreased the expression of Myostatin and apoptotic markers in myogenic cells *in vitro*. Consistent with these observations, Magic-Factor 1 promoted survival and differentiation (Cassano et al., 2008). Moreover, transgenic mice expressing muscle-specific Magic-factor 1 displayed hypertrophic fast twitch fibers with increased endurance to exercise, in addition to partially rescuing the degeneration observed in *α-sarcoglycan* KO mice (Cassano et al., 2008). Thus, tissue-specific engineered proteins hold potent clinical applications for ameliorating muscle wasting associated with dystrophies.

In addition to modulating specific signaling pathways, exercise and electrical stimulation appear to be promising therapeutic approaches in countering atrophy. Exercise has been proven to be effective in reducing muscle loss and in mobilizing SCs in aging muscles (Snijders et al., 2009). Concomitant to the increase in SC content, an up-regulation of myogenic regulatory factors and a reduction in Myostatin expression have been observed (Snijders et al., 2009). The beneficial effects of exercise have also been reported in disuse atrophy occurring with immobilization, and correlate with an increase in IGF-1 and Myogenin, and a decrease in Myostatin levels (Adams et al., 2007). Recent reports have demonstrated that the decrease in muscle size and SC number occurring during hind limb unloading could also be attenuated by electrical stimulation (Zhang et al., 2010b; Guo et al., 2012; Dirks et al., 2014). Although a causal relationship between SC activation and an attenuation in atrophy remain to be fully established, it seems that electrical stimulation can modify SC activity and prove beneficial to other forms of atrophy such as sarcopenia (Kern et al., 2014).

Another promising approach to ameliorate loss of muscle mass is cell therapy. Transplantation of SCs and other cellular types capable of myogenic differentiation have been shown to improve muscle phenotypes associated with different primary genetic diseases, especially in muscular dystrophies. In most of these studies, the rational of the cell therapy approach involves using cells to carry therapeutic genes into myofibers (Partridge and Davies, 1995). After transplantation, healthy donor-derived or patient-derived corrected cells fuse with existing myofibers and correct for the absence of expression from the mutated gene. The efficacy of this approach lies in the ability of the transplanted cells to not only differentiate into myofibers, but also to replenish the reservoir of stem cells and thereby sustain the repair process. Several studies have been exploring the potential of other stem cells besides SCs for therapeutic purposes (Peault et al., 2007). A complete and exhaustive discussion of these studies is beyond the scope of the present report and the readers are directed to comprehensive reviews that extensively describe cell transplantation approaches in primary genetic myopathies (Skuk and Tremblay, 2003; Price et al., 2007; Quattrocelli et al., 2010; Tedesco et al., 2010; Meng et al., 2011; Meregalli et al., 2013; Sirabella et al., 2013). We focus in this paragraph on acquired muscle wasting conditions. Recent observations suggest that a cell transplantation approach could be useful in reducing muscle loss after hind limb suspension and denervation (Plowman et al., 2014; Kim et al., 2015). Importantly, it has been reported that transplantation of SCs attached to their myofiber coupled with muscle injury could prevent the loss of muscle mass associated with aging (Hall et al., 2010). Intriguingly, transplantation in the absence of injury did not result in a similar increase in muscle mass (Hall et al., 2010). The interpretation of this observation is confounded by the high rate of lethality of donor cells generally associated with transplantation (Beauchamp et al., 1999). Nevertheless it supports the hypothesis that the authors of this study propose that unknown factors produced during injury can signal to fiber-associated SCs, and promote their long-term engraftment and hypertrophic function (Hall et al., 2010). In these set of experiments, fiber-associated SCs were transplanted into muscles of young mice and an increase in muscle mass was observed in 2-year-old mice (Hall et al., 2010). However, it remains to be tested whether heterologous transplantation of young fiber-associated SCs into injured muscles of old mice would also be beneficial in ameliorating sarcopenia. A large body of evidence indicates that during aging, local and systemic environments undergo profound changes that bear a negative impact on muscle precursor cell activity and regenerative potential (Gopinath and Rando, 2008), thus proving to be a major hurdle in transplantation approaches in aged muscles. Intriguingly, a recent study demonstrated that inhibiting p38α and p38β signaling transiently in myogenic progenitors isolated from aged mice, in conjunction with culture on soft hydrogel substrates rejuvenates their regenerative potential and increases muscle strength upon transplantation into damaged muscles of aged mice (Cosgrove et al., 2014). Although it is not clearly understood as to how these mechanisms enhance the regenerative capacity of old SCs, these observations suggest that the detrimental effects of an aging muscle microenvironment can be circumvented. Changes in the local and systemic environments are not a feature of aging alone, but are also profoundly altered in other chronic muscle wasting conditions, such as cancer cachexia (He et al., 2013). Future studies will be required to evaluate the efficacy of cell transplantation approaches in ameliorating muscle loss under various atrophic stimuli.

## Concluding Remarks

In conclusion, it is becoming increasingly evident that understanding the basic molecular mechanisms underlying various forms of atrophy is crucial to develop defined parameters and describe distinct features that categorize different forms of atrophy. It is also possible that different forms of atrophy could co-exist, such as disuse atrophy that accompanies aging coupled to bedridden conditions, as a result of chronic debilitating illnesses. Moreover, aging is a risk factor for many pathologies impacting muscle mass (Evans, 1995). Such multiple cause-related atrophies warrant the use of combinatorial therapies that selectively target the signaling pathway or pathways involved in specific atrophic processes. It has also become significantly clear that SC-focused studies expand the approaches that can be used to counter muscle loss. In general, despite a large body of evidence in favor of a role for SCs at least in some forms of atrophy, there have been reports that have restricted and thereby defined the window of SC action, or have evaluated SC functionality quantitatively rather than qualitatively. As such, these studies may have contributed to the “quasi-status” of SC involvement in atrophy. This, in addition to a knowledge of the molecular and cellular changes in SCs accompanying atrophy offer a powerful tool in being able to manipulate this compartment pharmacologically to increase muscle mass. In this context, SC-specific gene ablation studies in mice would provide a promising avenue to uncover novel signaling networks and thereby adopt a more focused approach towards tackling muscle wasting diseases in humans. These studies are currently being limited by difficulties in achieving a complete ablation of genes in a SC-specific manner, and the development of highly efficient strategies will be key in obtaining an accurate assessment of the requirement of the candidate genes during atrophy. A corollary to this approach is a comprehensive understanding of the systemic factors and the molecular milieu constituting the SC niche in the context of various atrophic conditions. In providing the means to modulate and eventually enhance SC function, this area of study renders itself to alternate therapeutic strategies such as nutritional interventions, other than exercise or pharmacological-based solutions (Alway et al., 2014). This is especially relevant in aging individuals with unrelated muscle wasting pathologies, wherein there are considerable limitations during the course of treatment. This shift in focus of examining molecular events during atrophy from fiber to SCs can potentially further be exploited even in scenarios that have reported limited involvement of the SC compartment in restoring muscle mass. A critical discussion of the cellular and molecular events operating in the atrophying muscles and SCs will contribute to the field for further studies investigating novel approaches to ameliorate muscle wasting diseases.

## Conflict of Interest Statement

The authors declare that the research was conducted in the absence of any commercial or financial relationships that could be construed as a potential conflict of interest.
